# Effect of Different Comonomers Added to Graft Copolymers on the Properties of PLA/PPC/PLA-g-GMA Blends

**DOI:** 10.3390/polym14194088

**Published:** 2022-09-29

**Authors:** Lixin Song, Qian Zhang, Yongsheng Hao, Yongchao Li, Weihan Chi, Fei Cong, Ying Shi, Li-Zhi Liu

**Affiliations:** 1 Advanced Manufacturing Institute of Polymer Industry, Shenyang University of Chemical Technology, Shenyang 110142, China; 2 College of Materials Science and Engineering, Shenyang University of Chemical Technology, Shenyang 110142, China

**Keywords:** poly (lactic acid), polypropylene carbonate, PLA-g-GMA graft copolymer, comonomer, grafting degree

## Abstract

The melt-free radical grafting of glycidyl methacrylate (GMA) onto poly (lactic acid) (PLA) with styrene (St), α-methylstyrene (AMS), and epoxy resin (EP) as comonomers in a twin-screw extruder was used to prepare PLA-g-GMA graft copolymers. The prepared graft copolymers were then used as compatibilizers to prepare PLA/PPC/PLA-g-GMA blends by melt blending with PLA and polypropylene carbonate (PPC), respectively. The effects of different comonomers in the PLA-g-GMA graft copolymers on the thermal, rheological, optical, and mechanical properties and microstructure of the blends were studied. It was found that the grafting degree of PLA-g-GMA graft copolymers was increased to varying degrees after the introduction of comonomers in the PLA-g-GMA grafting reaction system. When St was used as the comonomer, the grafting degree of the PLA-g-GMA graft copolymer increased most significantly, from 0.8 to 1.6 phr. St as a comonomer also most improved the compatibility between PLA and PPC, and the haze of the blends was reduced while maintaining high transmittance. In addition, the PLA-g-GMA graft copolymer with the introduction of St as a comonomer significantly improved the impact toughness of the blends, while the thermal stability and tensile strength of the blends remained largely unchanged.

## 1. Introduction

It is anticipated that, in the coming decades, biodegradable polymers will gradually replace traditional non-degradable plastics. Polylactic acid (PLA) is a biodegradable and biocompatible thermoplastic with a high modulus, high strength, high transparency, and excellent processing properties [1,2,3], which has led to it being widely used in the research of the modification of biodegradable materials. However, PLA has some inherent weaknesses, such as poor ductility and low tensile elongation at break [4,5], which greatly restricts the application of PLA in various fields. To eliminate this disadvantage, researchers have toughened PLA by blending it with other flexible and biodegradable polymers that exhibit lower glass transition temperatures (T_g_) [6], such as polybutylene terephthalate (PBAT) [7,8,9], polycaprolactone (PCL) [10,11], polypropylene carbonate (PPC) [12,13] and polybutylene succinate (PBS) [14]. PPC is of particular interest as it is the only one of the above materials synthesized from the “greenhouse gases” carbon dioxide (CO_2_) and propylene oxide (PO), which not only reduces the dependence on oil resources but also effectively alleviates the greenhouse effect [15,16]. PPC also possesses good properties such as toughness, biodegradability, and translucence [17,18], so PPC and PLA were complementary in performance [19]. However, while PPC and PLA have a degree of compatibility due to their similar chemical structures, many studies have revealed that this compatibility is low, and the properties of the blended materials cannot meet the actual use requirements. Thus, it is necessary to find a simple and effective method to improve the compatibility of PLA/PPC blends to improve their mechanical properties.

Reactive compatibilization is a simple and effective method to improve the interfacial interaction between the two phases of blends. The reaction group contained in the reactive compatibilizer can generate block or graft copolymer between different polymer interfaces in the process of blending. This block or graft copolymer is similar to coupling agent or emulsifier, which can reduce the interfacial tension between the two phases of blending. Due to the decrease in interfacial tension, the molecular chains in the two phases can penetrate each other through the interface, which increased the thickness of the interface layer and the interfacial adhesion, thus greatly improving the physical properties of blend material. This technique has been shown to provide a better dispersion and more uniform phase morphology by chemical reaction during melt blending [20]. Graft polymers of maleic anhydride (MAH) [21] and glycidyl methacrylate (GMA) [22] are commonly used as reactive compatibilizers in polyester blends to improve their compatibility by forming strong chemical bonds between the blends. MAH is less reactive due to its symmetrical structure and low electron density around the carbon–carbon double bond [23], whereas GMA has a bifunctional character: a free radically reactive double bond and an epoxy group that can react with other functional groups (carboxyl, hydroxyl, anhydride, and amine) [24,25,26,27], making it easier for GMA to react with free radicals [28]. In addition, MAH graft copolymers can stain materials yellow, which has a certain impact on the optical properties of the material and have an irritating odor. This has led to GMA graft copolymers being more commonly used to improve the compatibility of blends. During the melt-blending process, the epoxy group in GMA and the polyester hydroxyl or carboxyl group form a copolymer between the interface of the two phases through a nucleophilic substitution reaction [29], which reduces the interfacial tension and enhances the bonding force between the two phases. However, GMA is not active toward macroradicals, so the melt-free radical grafting reactivity and grafting degree of GMA graft copolymer are low. The presence of an initiator (peroxide) during the grafting reaction also leads to the degradation of the material and is accompanied by side reactions such as cross-linking and chain breaking [30]. Many approaches have been tried to improve the grafting degree of monomers and inhibit side reactions during the grafting reaction, one of the simplest and most effective of which is the introduction of a comonomer. Luo et al. [31] used a reactive extrusion method to melt graft MAH and α-methylstyrene (AMS) onto polypropylene (PP) and found that the addition of AMS as a comonomer can effectively improve the MAH grafting degree and effectively reduce the degradation of PP molecules. Gong et al. [32] studied GMA-grafted isobutylene-isoprene rubber (IIR) (IIR-g-GMA) copolymers and GMA/styrene (St) grafted IIR (IIR-g-St-GMA) copolymers and found that, compared with IIR-g-GMA, IIR-g-St-GMA showed a significantly higher grafting degree and effectively improved the compatibility of the IIR and polyamide 12 (PA12) copolymers. Lou et al. [33] grafted MAH and epoxy resin (EP) onto PP and added a certain amount of tetramethylthiuram disulfide (TMTD). They found that the addition of additional EP in the reaction system could reduce the sublimation of MAH and improve the graft reaction of MAH, while the addition of TMTD was conducive to the reaction. They also found that the mechanical properties of the composites were significantly improved by adding PP-g-MAH containing EP to PP/glass fiber (GF) composites.

Based on the above analysis, we selected a PLA-g-GMA graft copolymer as the reactive compatibilizer for the blending system of PLA and PPC in this work. On the one hand, PLA-g-GMA graft copolymer contained easily reactive functional groups that can react with PLA/PPC blends, which was particularly suitable for compatibilization between PLA and PPC with poor compatibility. Compared with non-reactive compatibilizers, PLA-g-GMA graft copolymer has the advantages of less dosage, low cost and good compatibilization effect. Therefore, it plays an important role in PLA blending research. We prepared PLA graft copolymers by the melt grafting of GMA onto PLA with the addition of St, AMS, and EP as comonomers. The prepared graft copolymers were then melt blended with PLA and PPC to prepare PLA/PPC/PLA-g-GMA blends. The effects of the different comonomers in the PLA-g-GMA graft copolymers on the thermal, rheological, optical, and mechanical properties of the blends were investigated, and the microscopic morphology of the blends was also studied.

## 2. Material and Methods

### 2.1. Materials

Polylactic acid (PLA) in pellet form with a density of 1.24 g/cm^3^ and a melt index of 7 g/min is supplied by Nature Works, USA, as 4032D. Polypropylene carbonate (PPC) is a solid particle, with a density of 1.28 g/cm^3^ and a melt index of 8 g/min, which is supplied by Boda Orient New Chemical Company, China, as BD-211. Glycidyl methacrylate (GMA) (analytical pure) is a liquid with content ≥ 99%, which is provided by Dow Chemicals, USA. Dicumyl peroxide (DCP) (chemically pure) is granular, with a purity of 99% and a melting point of 41.3 °C, which is provided by Chengdu Best Reagent Co., Ltd., Chengdu, China. Styrene (St) (chemically pure), a colorless and transparent liquid with 99% content and 0.909 g/mL density, is supplied by Tianjin Damao Chemical Reagent Plant, Tianjin, China. α- Methyl styrene (AMS) (chemically pure), a colorless transparent liquid, with a content of 99% and a density of 0.911 g/cm^3^, is provided by Sinopharm Chemical Reagent Company, Shanghai, China. Epoxy resin (EP) is a colorless viscous liquid with a viscosity of 14,000 and effective substance ≥ 99%, which is provided as E51 by the Shanghai Resin Factory Company, Shanghai, China.

### 2.2. Preparation of the Graft Copolymers

For the functionalization of PLA, DCP was used as the initiator. The DCP was dissolved in a mixed solution of GMA and acetone, to which was added the appropriate amount of St, AMS, and EP, respectively. The mixtures were then mixed well with PLA at a certain mass ratio. After the acetone had completely volatilized, PLA-g-GMA and other graft copolymers were prepared using a twin-screw extruder. The specific composition of the graft copolymers is shown in Table 1. It should be noted here that previous studies have shown that PLA-g-GMA graft copolymers with a GMA content of 3 phr achieve the best properties of the PLA/PPC blends [34]. The temperature of the twin-screw extruder was set to 165, 175, 175, 175, 175, 175, and 160 °C, respectively, from the feeding area to the mold, i.e., I, II, III, IV, V, VI and the die head, and the screw speed was set to 70 rpm. The extruded rods were cooled in a water bath and then granulated before being dried in a vacuum oven at 60 °C for 12 h.

### 2.3. Purification of the Graft Copolymers

About 2 g of each PLA-g-GMA graft copolymer was weighed, heated and refluxed in 25 mL of trichloromethane for 1 h, then precipitated and washed, and filtered in acetone three times to remove oligomers and unreacted monomers. The purified samples were dried under a vacuum at 70 °C for 12 h.

Chemical titration was used to determine the grafting degree (G_d_) of GMA onto PLA [35]. First, 0.2 g of the purified sample was dissolved in tetrahydrofuran, and then 0.05 mol/L HCL-isopropanol standardized solution was added, and the mixture was reflux heated for 0.5 h. Phenolphthalein was used as the indicator. The prepared solution was titrated with 0.05 mol/L NaOH-ethanol standardized solution, and then the grafting degree (G_d_) of the graft copolymer was determined using Equation (1).

(1) $$D_{g}=\frac{N\;\times \left(V_{0}-V\right)\times 142.5}{1000\;W}$$

where V_0_ is the volume of NaOH-ethanol standardized solution required to neutralize pure PLA (mL), V is the volume of NaOH-ethanol standardized solution required to neutralize the graft copolymer (mL), N is the molar concentration of the NaOH-ethanol standard solution, 142.15 is the molecular weight of GMA, and W is the weight of each graft copolymer sample (g). Grafting efficiency (G_e_) was the ratio of the mass of GMA involved in grafting to the total mass of GMA added.

### 2.4. Preparation of the Blends

The thermal stability of pure PPC was poor, and a large amount of thermal degradation occurred during the melt processing at 150 °C [36]. Using maleic anhydride (MAH) to react with PPC can slow down the thermal degradation of PPC. MAH can be partially grafted onto the terminal hydroxyl group of PPC to generate PPC-MA, which can effectively inhibit the degradation of PPC chain degradation and improve the thermal stability of PPC. Zhou et al. found that the initial decomposition temperature of PPC was increased from 150 to 200 °C, and the thermal stability of PPC-MA was enhanced by thermogravimetric analysis (TGA) [37].

Before the experiment, PLA and PLA-g-GMA graft copolymers were dried in an oven at 60 °C for 12 h, while PPC was dried in an oven at 35 °C for 12 h. After drying, PLA, PPC and PLA-g-GMA graft copolymers are melt blended in a twin-screw extruder to prepare PLA/PPC/PLA-g-GMA blends (see Table 2 and Table 3 for the specific composition and Section 2.2, above, for extrusion parameters). The extruded rods were cooled in a water bath and then granulated before being dried in an oven at 60 °C for 12 h. Finally, WZS10D micro injection molding machine was used for injection molding to prepare Izod impact test and tensile test samples. The barrel temperature was 180 °C, the mold temperature was 25 °C, the injection pressure was 0.6 MPa, and the pressure holding time was 10 s. The preparation standard of tensile test samples was GB/T 1040-1BA, and the preparation standard of Izod impact test samples was GB/T 1843/1-A.

### 2.5. Characterization

- Fourier transformation infrared spectroscopy (FTIR) analysis: The purified and dried PLA-g-GMA graft copolymers were molded into a film on a plate vulcanizer (400X400X2, Qingdao Yadong Rubber Machinery Co., Ltd., Qingdao, China) and analyzed by FTIR. FTIR spectra were recorded on a NEXUS 470 PC. The scanning range of infrared test was 7400~350 cm^−1^, the resolution was 0.5 cm^−1^, the wave number accuracy was 0.01 cm^−1^, and the number of scans was 75 times/s.
- Nuclear magnetic resonance analysis (^1^H-NMR) analysis: Weigh about 15 mg of PLA and PLA-g-GMA graft copolymer. Deuterated chloroform (CDCl_3_) and tetramethylsilane (TMS) were used as solvent and the internal standard, respectively. Bruker Ascend 500 ⅠⅠⅠ magnetic resonance analyzer was used for testing, scanning 128 times.
- Differential Scanning Calorimetry (DSC) analysis: Differential scanning calorimetry analysis (DSC, TA Q200, TA, New Castle, DE, USA) was performed by weighing approximately 5 mg of the PLA/PPC/PLA-g-GMA blends sample into a crucible. The temperature was raised from 0 to 200 °C at a rate of 10 °C/min and kept constant for 5 min to eliminate thermal history, which was further lowered to 20 °C at the same rate. The gas flow was 2.5 L/min.
- Thermogravimetric (TGA) analysis: Thermogravimetric analysis (TGA, STA449C, NETZSCH, Selb, Germany) was performed under flowing nitrogen (100 mL/min) at a heating rate of 10 °C/min. Approximately 15 mg of PLA/PPC/PLA-g-GMA blends were heated from room temperature to 500 °C.
- Rheological analysis: Rheological measurements of the blends were on a Dynamic mechanics analyzer (DHR-2, TA, New Castle, DE, USA). Frequency sweep for the PLA/PPC/PLA-g-GMA blends was under nitrogen using 25 mm plate geometry. The gap distance between the parallel plates was 0.8 mm. The sheet blends were about 1.0 mm in thickness. The angular frequency range used during testing was 0.1–100 rad/s with a shear strain of 1.0%. The temperature was plotted at 180 °C.
- Melt Flow Rate (MFR) analysis: The MFR was determined by a melt flow rate instrument (GT-7100-MH, Gotweil Scientific Instruments Co., Ltd., Qingdao, China) at 190 °C with a load of 2.16 kg (test standard was ASTM D1238-2013), MFR is calculated according to Equation (2):
  (2) $$\mathrm{MFR}=\frac{600\;m}{t}\;$$
  where m was the average mass of the material (g) and t was the interval sampling time (10 s).
- Optical property: The PLA/PPC/PLA-g-GMA blends were pressed into films of approximately 80 μm thickness using a flat vulcanizer and tested for haze and transmittance using a CS-700 haze meter (Hangzhou Caipu Technology Co., Ltd., Hangzhou, China).
- Mechanical property: According to GB/T1843/1-A, Izod notch impact strength was determined with a GT-7045-MD impact tester (Songshu Instrument Co., Ltd., Dongguan, China), the pendulum used was 2.750 J. The tensile properties were measured according to GB/T1040-1BA using a tensile testing machine (Instron 3365, Instron, Boston, MA, USA) at a crosshead speed of 25 mm/min. The test was performed at room temperature, and the average values of at least five tests were reported.
- Scanning electron microscopy (SEM): The fracture surface of the PLA/PPC/PLA-g-GMA blends were sputter-coated with gold and then observed using a scanning electron microscope (JSM-5600LV, JEOL, Tokyo, Japan).

## 3. Results and Discussion

### 3.1. FTIR and ^1^H-NMR Analysis

**Figure 1:**
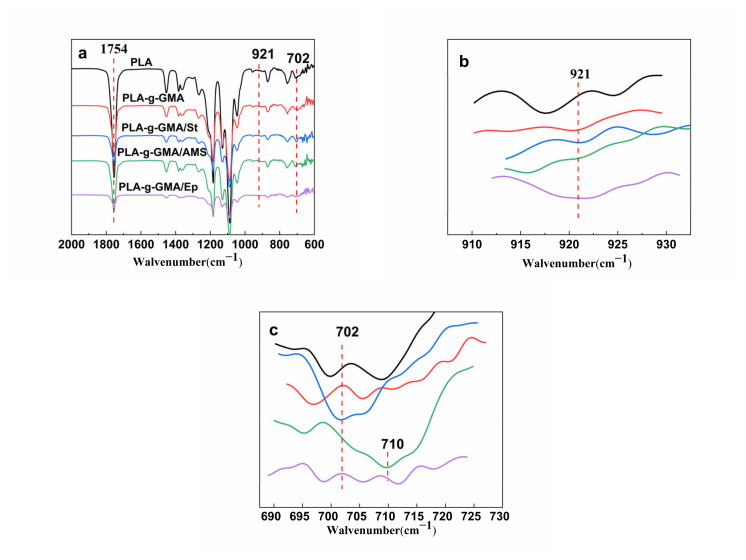
FTIR spectra of pure PLA and the PLA-g-GMA graft copolymers (**b**,**c**) are partial enlarged views of the red-dashed areas in (**a**).

Figure 1 shows the FTIR spectra of the purified PLA/PPC/PLA-g-GMA blends. The peak at 1754 cm^–1^ in all spectra was the characteristic absorption peak of carbonyl C=O in the PLA backbone. Comparing the FTIR spectra of PLA and the PLA-g-GMA graft copolymers, it can be seen that the spectra of all samples were approximately the same (Figure 1a), differing only in that the graft copolymers had a new signal peak at 921 cm^–1^, not present in the PLA spectrum, which could be assigned to the asymmetric stretching vibration of the epoxy group in the GMA structure (Figure 1b). In the PLA-g-GMA/St spectrum, an absorption peak of the benzene ring was observed at about 702 cm^–1^ [38], while the PLA-g-GMA/AMS graft copolymer had a new absorption peak at 710 cm^–1^, which belonged to the asymmetric stretching vibration of the AMS characteristic phenyl group (Figure 1c) [35]. When EP was used as a comonomer, the intensity of the epoxy group peaks in the spectrum was significantly improved due to the introduction of the epoxy group-containing EP in the grafting reaction system. The findings of the FTIR analysis showed that GMA, St, AMS, and EP had all been successfully grafted onto PLA. In addition, no absorption peaks corresponding to carbon–carbon double bonds (around 1630 cm^–1^) were found in the spectra of any of the graft copolymers, indicating that the unreacted monomers were completely removed during the purification process.

**Figure 2:**
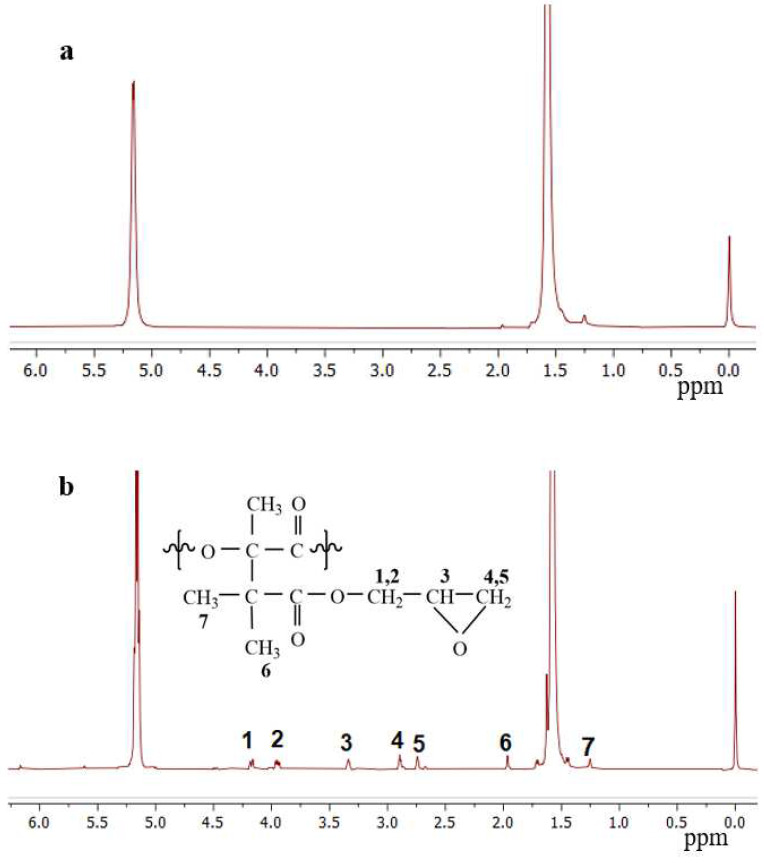
^1^H-NMR spectra of PLA (**a**) and PLA-g-GMA graft copolymer (**b**).

Figure 2 showed the ^1^H-NMR spectra of PLA and PLA-g-GMA, which can also prove that GMA is successfully grafted onto PLA. From Figure 2a,b, two peaks were observed at 5.2 and 1.6 ppm in the ^1^H-NMR spectra of PLA and PLA-g-GMA. The peak at 5.2 ppm represented methine protons and peaks at 1.6 ppm represented methyl protons. However, compared to the spectra of PLA (Figure 2a) and PLA-g-GMA (Figure 2b), new and weaker peaks were observed at 1.1–4.4 ppm, which represented the GMA constitutional unit of proton CH, CH_2_ and CH_3_ (see inset of the corresponding protons of 1–7). The new multipeak confirmed that GMA was successfully grafted onto the PLA (Figure 2) [39].

### 3.2. Characterization of the Graft Copolymers

**Figure 3:**
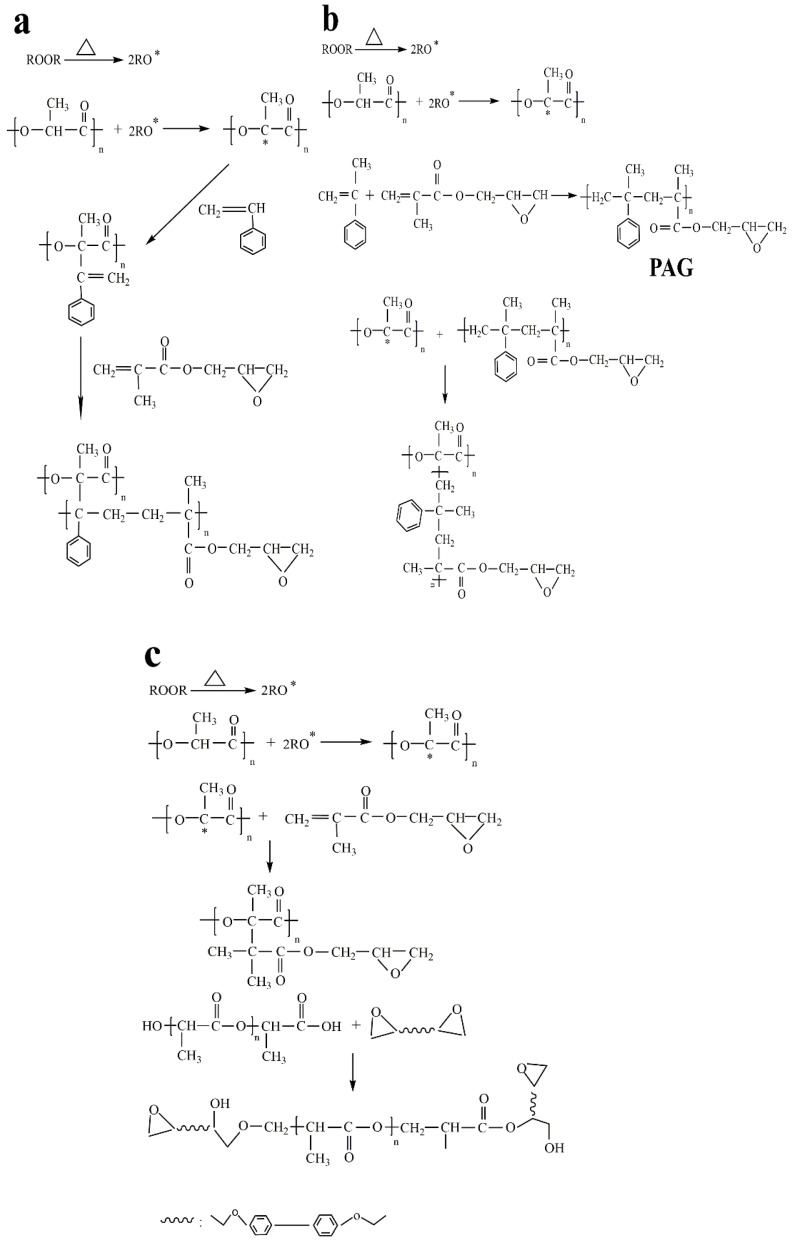
Reaction mechanisms: (**a**) PLA-g-GMA/St, (**b**) PLA-g-GMA/AMS, and (**c**) PLA-g-GMA/EP.

Table 4 showed the grafting degree (G_d_) and grafting efficiency (G_e_) of PLA-g-GMA graft copolymers containing different comonomers. As can be seen, the grafting degree of the PLA-g-GMA graft copolymers all increased to different degrees after the introduction of the different comonomers in the grafting reaction system, increasing 2 times, 1.6 times, and 1.1 times when St, AMS, and EP, respectively, were used as the copolymers. This was mainly due to the competition between grafting and chain scission (β-scission) of the PLA macro radicals in a single monomer grafting system. The PLA macro radicals underwent more extensive β-scission due to the ‘‘bite’’ reaction with the terminal hydroxyl (–OH), or carboxyl, group (–COOH), resulting in a low grafting degree of PLA-g-GMA graft copolymers. However, in the dual-monomer grafting system, the carbon–carbon double bonds (C=C) in St were more reactive than those in GMA. When St was added as a comonomer in the melt grafting system, the conjugated double bond of St enabled it to react rapidly with PLA macromolecular radicals to form stable styryl macromolecular radicals. GMA copolymerizes easily with styryl macromolecular radicals, and the reaction rate of this reaction was greater than that of the reaction with GMA with PLA macromolecular radicals. Similarly, AMS can form AMS-co-GMA (PAG) oligomers with GMA, which then react with PLA macromolecular free radicals to graft GMA onto the main chain of PLA through PAG, which improves the grafting degree of the PLA-g-GMA graft copolymer. It is well known that the copolymerization of two monomers is determined by the reactivity ratio of the monomers. The Q-e scheme has been developed to determine the ratios for each pair of monomers in a particular copolymerization [27]:

(3) $$r_{1}=\left(\frac{Q_{1}}{Q_{2}}\right)\exp\left[{-e}_{1}\left(e_{1}{-e}_{2}\right)\right]$$

(4) $$r_{2}=\left(\frac{Q_{2}}{Q_{1}}\right)\exp\left[{-e}_{2}\left(e_{2}{-e}_{1}\right)\right]$$

where Q_1_ and Q_2_ are measures of the reactivity of monomers and related to the resonance stabilization of the monomer, and the constants e_1_ and e_2_ are measures of the polarity of the monomers. According to the Q-e scheme, copolymerization will proceed more suitably between monomers with similar Q values. The Q values of GMA, St, and AMS are 0.98, 1.00, and 0.53, respectively. Therefore, GMA was more likely to copolymerize with styrene macromolecular radicals, which was the reason for the significantly higher GMA grafting degree when St was used as a comonomer [30,40]. When EP was used as a comonomer, the chemical reactions in the grafting reaction system were relatively complex. The epoxy group in EP reacted with the hydroxyl or carboxyl group of PLA to form the PLA-g-EP graft copolymer. When EP was used as a comonomer, the grafting degree of PLA-g-GMA increased by 0.1 phr, which was probably due to the part containing the epoxy group in the PLA-g-EP graft copolymer. Therefore, EP did little to improve the grafting degree of PLA-g-GMA graft copolymer, and PLA-g-EP graft copolymer could not effectively play the role of compatibilization [41]. The reaction mechanisms of the three comonomers are shown in Figure 3.

### 3.3. Optimum Content of the Graft Copolymer

**Figure 4:**
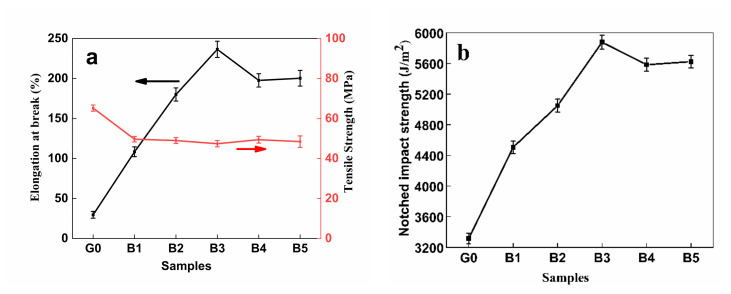
Effect of PLA-g-GMA content on mechanical properties of PLA/PPC/PLA-g-GMA blends (G0: PLA; B1: PLA/PPC; B2: PLA/PPC/PLA-g-GMA (5 phr); B3: PLA/PPC/PLA-g-GMA (10 phr); B4: PLA/PPC/PLA-g-GMA (15 phr); B5: PLA/PPC/PLA-g-GMA (20 phr)).

**Figure 5:**
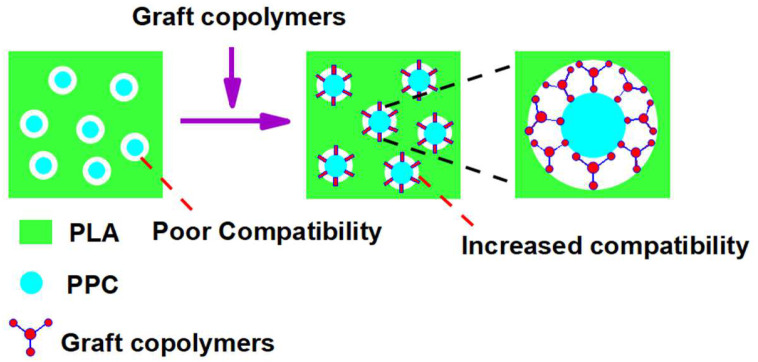
The compatibilization effect of PLA-g-GMA graft copolymer on PLA/PPC blends.

Figure 4 shows the variation in elongation at break, impact strength and tensile strength of the PLA/PPC/PLA-g-GMA blends with PLA-g-GMA graft copolymer content. Table 5 shows the relevant mechanical property data. Figure 5 shows the principle of PLA-g-GMA graft copolymer for compatibilization of PLA/PPC blends.

It can be seen from Figure 4 and Table 5 that the elongation at break and impact strength of pure PLA are 29.58% and 3315.4 J/m^2^, respectively. After PPC and PLA are blended, the elongation at break and impact strength of PLA/PPC blends are improved to a certain extent. In order to further improve the performance of the blends, PLA-g-GMA graft copolymer is introduced as a reactive compatibilizer in the blending process. From Figure 4 and Table 5, it can be found that when the content of graft copolymer was 10 phr, the elongation at break and impact strength of the blends increased to 236.46% and 5876.90 J/m^2^, respectively. Previous studies have found that the mechanical properties of such materials are influenced by interfacial strength [42]. When PLA-g-GAM graft copolymers were introduced into the blends (Figure 5), the copolymer sat between PLA and PPC, which enhanced the interaction force between the molecular chains at the interface of the two phases and, to a degree, improved the compatibility between the two materials [39], which in turn improved the elongation at break and impact strength of the blends. Continuing to increase the content of PLA-g-GMA graft copolymer in the system (15 or 20phr) led to the elongation at break and the impact strength of the blends decreasing, which may be because excess PLA-g-GMA graft copolymers can form multilayers of macromolecules located at the interface between the PPC and PLA matrix, causing self-entanglement among the PLA-g-GMA graft copolymers, rather than the PLA matrix, which affected the mechanical properties of the blends to a certain extent [43]. In addition, the tensile strength of the blends remained almost unchanged after the introduction of PLA-g-GMA graft copolymer into the blends (Figure 4a), so the introduction of PLA-g-GMA graft copolymer improved the toughness of the blends without affecting their rigidity.

In conclusion, the mechanical properties of the PLA/PPC/PLA-g-GMA blends were best when the PLA-g-GMA graft copolymer was 10 phr of the blends. Therefore, in subsequent experiments, the content of graft copolymer was kept as 10 phr.

### 3.4. DSC Analysis

**Figure 6:**
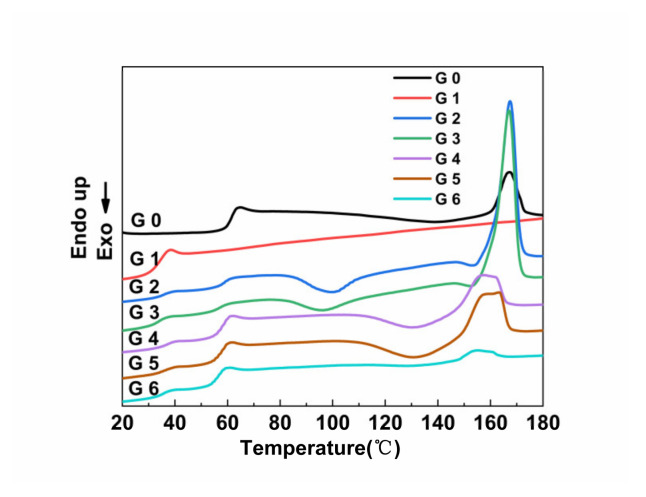
DSC curves of PLA, PPC, PLA/PPC and the PLA/PPC/PLA-g-GMA blends. (G0: PLA; G1: PPC; G2: PLA/PPC; G3: PLA/PPC/PLA-g-GMA; G4: PLA/PPC/PLA-g-GMA/St; G5: PLA/PPC/PLA-g GMA/AMS; G6: PLA/PPC/PLA-g-GMA/EP).

Figure 6 shows the DSC secondary warming curves of PLA/PPC/PLA-g-GMA blends, and Table 6 shows the related data. The blends showed heat absorption signal peaks at around 60 and 36 °C, which correspond to the glass transition temperatures (T_g_) of the PLA and PPC phases, respectively. As Table 6 shows, after introducing PLA-g-GMA graft copolymer into the blends, the ΔT_g_ decreased from 22.98 to 22.05 °C, which indicated that the compatibility between PLA and PPC was improved, but the ΔT_g_ only decreased by 0.93 °C. The ΔT_g_ of the blends was further reduced when the copolymer monomer was introduced into the PLA-g-GMA graft copolymer. When St was used as the copolymer monomer, ΔT_g_ decreased from 22.98 °C to 20.42 °C; when AMS was used as the copolymer monomer, ΔT_g_ decreased from 22.98 °C to 20.96 °C, a decrease of 2.56 and 2.02 °C, respectively. When EP was used as the comonomer, the ΔT_g_ decreased to 21.05 °C, a decrease of only 1.93 °C. This phenomenon is due to the grafting degree of the PLA-g-GMA graft copolymer, as shown in Table 2. When St was used as a comonomer, the PLA-g-GMA graft copolymer contained more epoxy groups, which reacted with the end groups of PLA and PPC, enhancing the interfacial adhesion between PLA and PPC, resulting in the improvement of the compatibility between PLA and PPC.

Table 6 also shows that the melting temperature (T_m_) and crystallinity (X_c_) of the blends gradually decreased after the graft copolymer-containing comonomers were introduced into the blends. This was because the reaction point worked as a physical cross-linking point, and the entangled structures in the amorphous area impeded the transportation of macromolecular chains between polymer chains, restricting the ability of PLA to crystallize and form a more amorphous phase, so the crystallinity of the blends was reduced [29]. In addition, the introduction of the three comonomers (St, AMS, EP) also led to longer branched chains forming in the blends, and the site-resistance effect of these branched chains depressed the tight stack of PLA and PPC chains, resulting in the reduction in the T_m_ of the blends [31].

### 3.5. TGA Analysis

**Figure 7:**
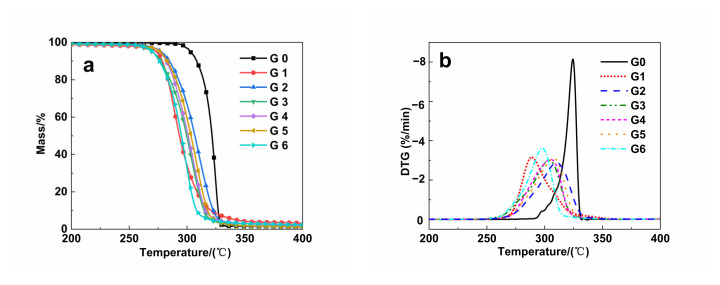
Thermogravimetric curves (**a**): TG curves, (**b**): DTG curves) of PLA, PPC, PLA/PPC and PLA/PPC/PLA-g-GMA blends (G0: PLA; G1: PPC; G2 PLA/PPC; G3: PLA/PPC/PLA-g-GMA; G4: PLA/PPC/PLA-g-GMA/St; G5: PLA/PPC/PLA-g-GMA/AMS; G6: PLA/PPC/PLA-g-GMA/EP).

Figure 7a,b shows the TG and DTG curves of pure PLA, pure PPC, PLA/PPC and PLA/PPC/PLA-g-GMA blends under nitrogen flow, respectively, and Table 7 shows the characteristic parameters of the TG curve of the blends. It can be seen from Figure 7 that the thermal stability of PLA was relatively good, while the thermal stability of PPC was relatively poor. After PLA and PPC were blended, the thermal stability of all blends was between pure PLA and pure PPC. The thermal stability of the blends was slightly decreased after the introduction of PLA-g-GMA graft copolymer in the blends, while the thermal stability of the blends was not further reduced but instead slightly increased after the introduction of the comonomers in the graft copolymers. It can be seen from Table 7 that the pure PLA/PPC blends had good thermal stability below 230 °C and did not decompose, and when the temperature exceeded 277.75 °C, the blends began to show slight decomposition. The weight loss rate reached the maximum at 310.25 °C, and the sample decomposed completely at 327.60 °C. After the introduction of the PLA-g-GMA graft copolymer into the blends, the T_5%_ of the blends decreased by 6.75 °C, and the thermal stability also decreased. This was probably because the preparation of the PLA-g-GMA graft copolymers was accompanied by degradation, cross-linking, and other side reactions, so the thermal stability of the blends was reduced to some extent after the graft copolymers were added to the blends. After the introduction of PLA-g-GMA graft copolymers containing different comonomer into the blends, the thermal stability of the blends did not further decrease but instead increased slightly. When St was used as a comonomer, the T_5%_ of the blends increased by 5.68 to 326.53 °C. This was because the conjugated double bond of St can rapidly react with PLA macromolecular free radicals, and the St radicals were more stable and had a longer half-life, which can better subsequent reactions with GMA, thus inhibiting PLA chain degradation, cross-linking, and other side reactions during the grafting reaction. When AMS was used as a comonomer, the T_5%_ of the blends increased by just 2.17 to 323.02 °C. Although AMS improved the grafting degree of PLA-g-GMA to some extent and increased the intermolecular forces between PLA and PPC, there are weak bonds in the AMS sequence, and the copolymers containing the AMS structure were prone to chain breakage, and under heating the blends depolymerized at their weak bonds and formed chain radicals, which could further initiate other chemical reactions [44,45], resulting in a smaller effect on T_5%_. When EP was used as a comonomer, the T_5%_ of the blends was 320.38 °C, which was close to that when no comonomer was introduced, which may be caused by the small effect of EP as a comonomer on the grafting degree of PLA-g-GMA graft copolymer. It can also be seen from Figure 7b that the temperature corresponding to the maximum decomposition rate of pure PLA was the highest, while the PPC was relatively low. The maximum decomposition rate of all the blends after the blending of PLA and PPC corresponds to a temperature somewhere in between. After the introduction of PLA-g-GMA graft copolymer into the blend, the T_max_ of the blends almost does not change significantly, which indicated that the PLA-g-GMA graft copolymer did not cause the blends to degrade significantly at a lower temperature.

### 3.6. Dynamic Rheological Analysis

**Figure 8:**
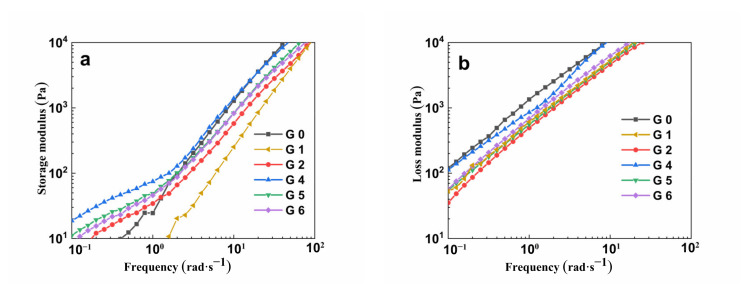

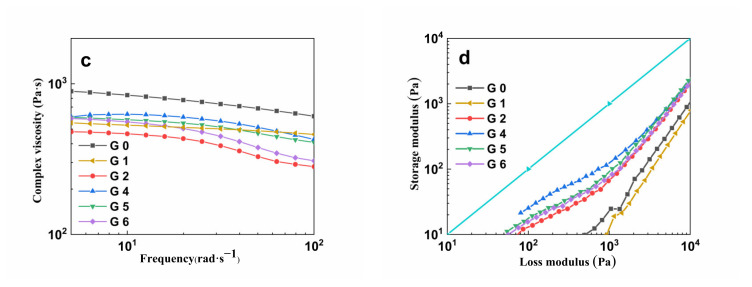
(**a**) Storage modulus, (**b**) loss modulus, (**c**) complex viscosity, and (**d**) Han curves of the PLA, PPC, PLA/PPC and PLA/PPC/PLA-g-GMA blends (G0: PLA; G1: PPC; G2: PLA/PPC; G4: PLA/PPC/PLA-g-GMA/St; G5: PLA/PPC/PLA-g-GMA/AMS; G6: PLA/PPC /PLA-g-GMA/EP; in (**d**): the straight line is diagonal).

Figure 8 shows the dynamic rheological analysis of pure PLA, pure PPC, PLA/PPC and PLA/PPC/PLA-g-GMA blends with different comonomers.

As Figure 8a,b show, after the introduction of the PLA-g-GMA graft copolymers containing comonomers in the blends, the storage modulus (G′) and loss modulus (G″) of the blends were higher than those of the PLA/PPC blends in the whole angular frequency test range (0.1~100 rad·s^–1^), and the higher the grafting degree of the PLA-g-GMA graft copolymer, the larger the G′ and G″ of the blends. The significant increase in G′ indicated that the elasticity of the blend melt was improved, resulting in enhanced interaction between PLA and PPC, and, thus, the compatibility of the blend was improved. In addition, after the introduction of the graft copolymers into the blends, the molecular weight of the blends became higher, and the friction resistance between the molecular chain segments gradually increased. Since the internal friction hinders the movement of the molecular chain, G″ increased.

As Figure 8c shows, the complex viscosity (η″) of all the blends exhibited a downward trend with increasing frequency, which is typical of the shear thinning behavior of the blends in the composites. Initially, the shear rate was low, the molecular chains were entangled with each other, and the movement between the chain segments was difficult, while with the acceleration of the shear rate, the molecular chains of the blends began to gradually orientate, and the friction between the chain segments decreased, which manifested as a decrease in viscosity on the macroscopic scale. In addition, the η″ of the blends increased after the introduction of the PLA-g-GMA graft copolymers containing different comonomers into the blends. It is known that an increase in the η″ of the blends generally occurs when there is either a specific interaction between the phases or chemical bonding between the blend components [46,47]. This indicated that the epoxy group from the graft copolymer had reacted with the end hydroxyl group from PLA or PPC to form ester or hydrogen bonds. Under the action of the two binding forces, the interfacial bonding force between PLA and PPC was further improved, thus limiting the movement of the molecular chain, which led to a decrease in melt fluidity and an increase in melt strength [48]. Contrastingly, the number of epoxy groups reacting with hydroxyl groups in the graft copolymer influenced the grafting degree of the graft copolymer, which meant that the higher the grafting degree, the more reactions occurred, the stronger the intermolecular forces, and the more significant the increase in the η″ of the blends. Therefore, when St was used as the comonomer, the increase in η″ of the blends was most obvious, followed by when AMS and EP were used.

Han plots, which consist of the energy storage modulus as the ordinate and the loss modulus as the abscissa, can be used to compare the differences between elastic and viscous properties, with linear viscoelastic data being the most sensitive to structural differences. The diagonal line (G′ = G″) divides the graph into two parts and defines the transition from viscous behavior (G′ < G″) to elastic behavior (G′ > G″). The Han plots of all blends are shown in Figure 8d, from which it can be seen that no curves cross the diagonal line, i.e., the whole system was still dominated by viscosity. If a cross-linked structure was present, the system would be more elastic than viscous, which would be indicated by the curve crossing the diagonal in the Han plots. Thus, it can be shown that there was no excessive cross-linked structure in the system [49].

### 3.7. MFR Analysis

**Figure 9:**
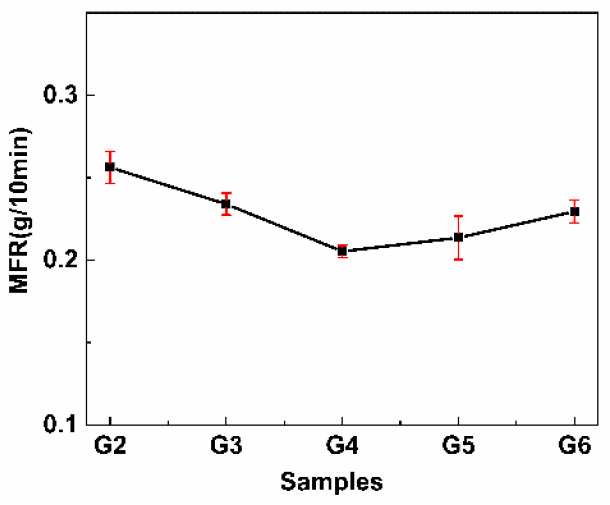
Melt index values of PLA/PPC and the PLA/PPC/PLA-g-GMA blends. (G2: PLA/PPC; G3: PLA/PPC/PLA-g-GMA; G4: PLA/PPC/PLA-g-GMA/St; G5: PLA/PPC/PLA-g-GMA/AMS; G6: PLA/PPC/PLA-g-GMA/EP).

MFR analysis was used to determine the melt flow rate of the PLA/PPC/PLA-g-GMA blends. The effect of different comonomers in the PLA-g-GMA graft copolymer on the MFR of the blends is shown in Figure 9.

As seen in Figure 9, the MFR values of the blends were reduced after the introduction of the PLA-g-GMA graft copolymers in the blends and were further reduced after the introduction of the comonomer in the PLA-g-GMA graft copolymers. This indicated that as the molecular weight of the blends increased, the melt flow rate decreased, which meant that the introduction of the comonomers inhibited the degradation of the blends to a degree. When St was the comonomer, the MFR of the blends reached its lowest value (0.2053 g/10 min), which can be attributed to the fact that St was more reactive than GMA, enhancing the grafting reaction between PLA and GMA, and reducing the β-breakage of PLA macromolecular radicals, thus inhibiting the degradation of PLA. When AMS was used as a comonomer, the MFR was 0.2136 g/10 min, which was slightly higher than that of St. This may be because AMS may preferentially graft onto the PLA backbone during the grafting reaction, hindering the grafting reaction between PAG and PLA macromolecular radicals, which would reduce the intermolecular forces between the blends and thus slightly increase the MFR of the blends compared with the value with St. When EP was used as a comonomer, the MFR was 0.2295 g/10 min, which was close to that when no comonomer was introduced into the PLA-g-GMA graft copolymer. This was probably because when EP was used as a comonomer, a certain amount of PLA-g-EP by-products were generated during the grafting process, which did not have a good compatibilization effect on PLA and PPC [41], so the intermolecular forces between PLA and PPC remained largely unchanged, which led to the MFR of the blends being close to that when the comonomer was not introduced.

### 3.8. Optical Properties

**Figure 10:**
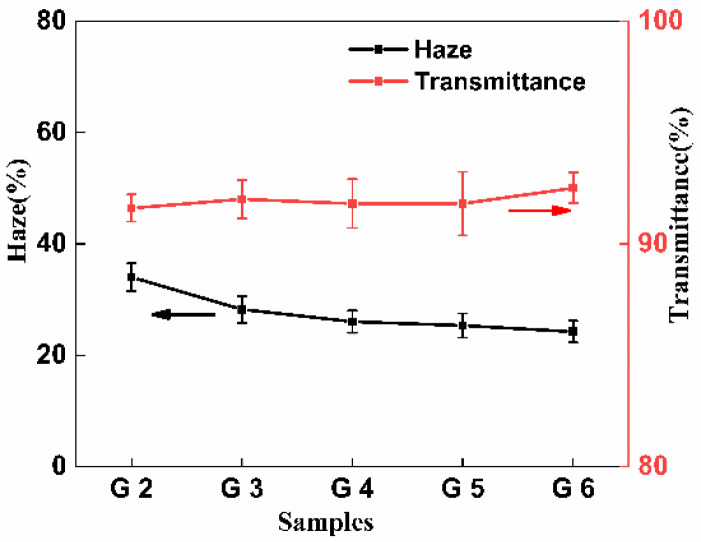
Effect of grafted monomers on the haze and transmittance of PLA/PPC and the PLA/PPC/PLA-g-GMA blends (G2: PLA/PPC; G3: PLA/PPC/PLA-g-GMA; G4: PLA/PPC/PLA-g-GMA/St; G5: PLA/PPC/PLA-g-GMA/AMS; G6: PLA/PPC/PLA-g-GMA/EP).

Figure 10 shows the effect of different comonomers in the PLA-g-GMA graft copolymers on the transmittance and haze of PLA/PPC/PLA-g-GMA blends films. It can be observed from Figure 10 that the haze of the pure PLA/PPC blends was 34.0%, and the transmittance was as high as 91.6%. After the introduction of the PLA-g-GMA graft copolymers in the blends, the haze of the blends decreased to 28.2%. It decreased further after the introduction of the comonomers in the PLA-g-GMA graft copolymers, being lowest when EP was used as the comonomer. This was mainly the influence of the degree of crystallization of the polymer, as, generally, the greater the internal crystallization, the greater the haze [50]. From the DSC analysis discussed above, it was clear that the crystallinity of the blends gradually decreased after the introduction of the comonomers in the PLA-g-GMA graft copolymers, and, hence, the haze of the blends also decreased. At the same time, the introduction of the graft copolymers did not significantly affect the transmittance of the blends, which all fluctuated in the range of 91.6~92.5%. These findings show that the introduction of the comonomer in the PLA-g-GMA graft copolymer can reduce the haze of the blends while retaining high transmittance, and the blends show relatively excellent optical properties as a whole.

### 3.9. Mechanical Properties

**Figure 11:**
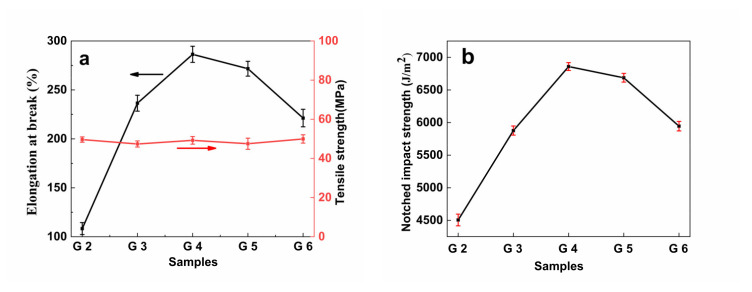
Effect of grafted monomers on the mechanical properties of PLA/PPC and the PLA/PPC/PLA-g-GMA blends; (G2: PLA/PPC; G3: PLA/PPC/PLA-g-GMA; G4: PLA/PPC/PLA-g-GMA/St; G5: PLA/PPC/PLA-g-GMA/AMS; G6: PLA/PPC/PLA-g-GMA/EP).

Figure 11 shows the effect of the different comonomers in PLA-g-GMA graft copolymers on the elongation at break, tensile strength, and impact strength of the PLA/PPC/PLA-g-GMA blends. Table 8 shows the relevant mechanical property data. It can be seen from Table 8 that the elongation at break and impact strength of PLA were 29.58% and 3315.4 J/m^2^, respectively, while the elongation at break of PPC was as high as 761.32%. It can be seen from Figure 11 that the toughness of PLA blends was improved after the blending of PPC. In order to further improve the mechanical properties of the blends, PLA-g-GMA graft copolymers were used as a reactive compatibilizers. After being introduced into the blend, the elongation at break of the blends further increased to 236.46%, and the impact strength increased to 5876.90 J/m^2^. The PLA-g-GMA graft copolymer improved the toughness of the blends to a degree, which was due to a small rise in the compatibility between PLA and PPC. After the introduction of the comonomers in the PLA-g-GMA graft copolymers, the elongation at break and impact strength of the blends first increased and then decreased. When St was used as the comonomer, the elongation at break and impact strength of the blends increased the most significantly (286.35% and 6858.6 J/m^2^, respectively), followed by AMS (271.67% and 6686.65 J/m^2^, respectively), while the mechanical properties of the blends when EP was used as the comonomer were close to those when no comonomer was introduced (221.25% and 5944.3 J/m^2^, respectively). This was because St can best enhance the grafting degree of PLA-g-GMA graft copolymer, and the higher the grafting degree, the more epoxy groups that can react with PLA or PPC end groups, and the better the interfacial compatibility between PLA and PPC. With the improvement of the compatibility of the blends, the physical entanglement of the molecular chains of the blends increased, the intermolecular forces were enhanced, and the interfacial tension was reduced. At the same time, the stress can be effectively transferred from the PLA phase to the PPC phase, and the toughness of the blends was improved. When AMS was used in place of St as the comonomer, its larger substituent size led to a larger spatial site resistance effect, so the reactivity with PLA macromolecular radicals was reduced, and the degree of improvement of the grafting degree of the PLA-g-GMA graft copolymer was reduced, resulting in the decrease in the degree of improvement of the elongation at break and impact strength of the blends. When EP was the comonomer, it did not promote the reaction between GMA and PLA but instead reacted directly with PLA to form PLA-g-EP graft copolymer, and the PLA-g-EP graft copolymer did not improve the compatibility between PLA and PPC, so it had little effect on the impact toughness of the blends. In addition, it can also be seen from Figure 11a that the tensile strength of the blends hardly changed when the three graft copolymers were introduced into the blends, and the value fluctuated around 49 MPa, which showed that the introduction of PLA-g-GMA graft copolymers containing different comonomers into the blends not only enhanced the toughness of the blends but also maintained their excellent rigidity, producing materials with excellent comprehensive properties.

**Figure 12:**
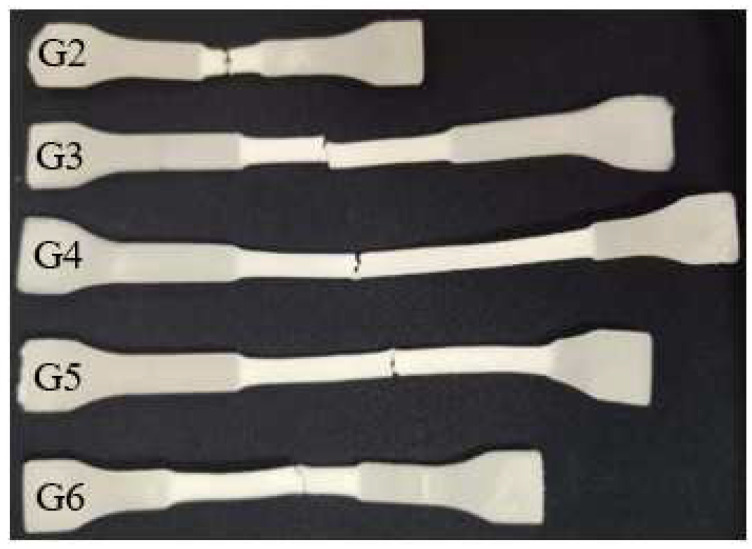
Tensile splines of the PLA/PPC/PLA-g-GMA blends (G2: PLA/PPC; G3: PLA/PPC/PLA-g-GMA; G4: PLA/PPC/PLA-g-GMA/St; G5: PLA/PPC/PLA-g-GMA/AMS; G6: PLA/PPC/PLA-g-GMA/EP).

Figure 12 shows the tensile test samples of the PLA/PPC/PLA-g-GMA blends, where the PLA-g-GMA graft copolymer contained different comonomers. It is obvious from the figure that the pure PLA/PPC was relatively brittle, while after the introduction of the PLA-g-GMA graft copolymer, the blends exhibited ductile extension. The extent of stress whitening further increased after the introduction of comonomers in the PLA-g-GMA graft copolymers, showing an obvious narrow neck and stress hardening. This change in the stress whitening zone of the blends was most obvious when St was used as the comonomer. The observed macroscopic trend in stress-whitening in the blends was consistent with the measured mechanical properties of the blends.

### 3.10. SEM Analysis

**Figure 13:**
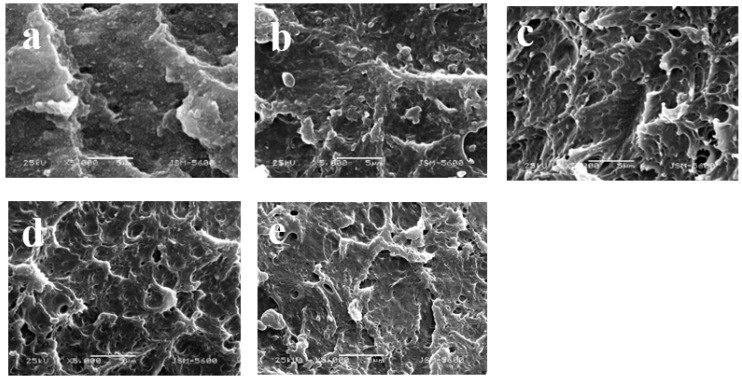
SEM images of the fracture surface in PLA/PPC and PLA/PPC/PLA-g-GMA blends ((**a**) PLA/PPC; (**b**) PLA/PPC/PLA-g-GMA; (**c**) PLA/PPC/PLA-g-GMA/St; (**d**) PLA/PPC/PLA-g-GMA/AMS; (**e**) PLA/PPC/PLA-g-GMA/EP).

To investigate the effect of different comonomers in PLA-g-GMA graft copolymers on the fracture morphology of the PLA/PPC/PLA-g-GMA blends, the impact cross-section of the blends was observed by SEM, as shown in Figure 13. The fracture surface of the pure PLA/PPC blends was relatively flat and smooth, and no obvious plastic deformation occurred (Figure 13a), which was due to the weak bonding force at the interface of PLA and PPC preventing effective transmission and dispersion of the stress between the two phases when subjected to external forces. When the PLA-g-GMA graft copolymer was added to the blends (Figure 13b), the impact fracture surface of the blends began to become rough and showed evidence of plastic deformation. When St was introduced as the comonomer in the PLA-g-GMA graft copolymer, the interface between PLA and PPC phases in the matrix was effectively strengthened, and the impact fracture surface of the blends showed an even rougher surface (Figure 13c), which was honeycomb, indicating that the PLA-g-GMA/St graft copolymer could effectively improve the compatibility of PLA and PPC and increase the entanglement and interfacial adhesion of the molecular chains of the PLA and PPC phases. The improved interfacial adhesion helped the material to generate a large plastic deformation zone along the fracture direction to absorb more energy and improve the impact strength of the blends [51,52]. When the comonomer was AMS (Figure 13d), the roughness of the impact fracture surface of the blends was slightly reduced compared with that of Figure 13c, but it also exhibited obvious honeycomb shape, showing ductile fracture characteristics. When EP was used as the comonomer (Figure 13e), the degree of roughness of the impact fracture surface of the blends was similar to that of Figure 13b. The experimental results show that the PLA-g-GMA/St graft copolymer could best improve the compatibility between PLA and PPC when St was used as the comonomer, as this changed the fracture mode of the blend from brittle fracture to ductile fracture. This was also consistent with the results of the tests of mechanical properties discussed above.

## 4. Conclusions

The addition of St, AMS, and EP as comonomer to the melt-grafting system of GMA onto PLA was to prepare PLA-g-GMA graft copolymers containing different comonomers. The prepared graft copolymers were melt blended with PLA and PPC to prepare PLA/PPC/PLA-g-GMA blends. The thermal, optical, and mechanical properties and micromorphology of these blends were extensively studied. The main conclusions can be summarized as follows:

FTIR and ^1^H-NMR analysis showed that GMA, St, AMS, and EP had been successfully grafted onto the PLA molecular chains to form PLA-g-GMA, PLA-g-GMA/St, PLA-g-GMA/AMS, and PLA-g-GMA/EP graft copolymers. After the introduction of the comonomer in the grafting reaction, the grafting degree of PLA-g-GMA graft copolymer was improved to varying degrees, and when St was used as the comonomer, the grafting degree was increased most significantly.

The introduction of PLA-g-GMA graft copolymers improved the toughness of PLA/PPC blends to the degree that depended on the graft copolymer content, with the elongation at break and impact strength of the blends reaching a maximum when the graft copolymer was present at 10 phr.

The PLA-g-GMA graft copolymers containing comonomers can further improve the compatibility between PLA and PPC; when St was used as the comonomer, the improvement effect was most obvious. At this time, the storage modulus, loss modulus, and complex viscosity of the blends were maximized without any negative effect on the thermal stability of the blends.

When the PLA-g-GMA graft copolymer contained comonomers, the blends film reduced the haze while maintaining high transmittance, reflecting good optical properties. Meanwhile, the elongation at break and impact strength of the blends were further improved, while the tensile strength remained almost unchanged. The elongation at break and impact strength of the blends reached the peak value (286.35% and 6858.6 J/m^2^, respectively) when St was used as a comonomer. At this time, the impact fracture mode of the blends showed obvious ductile fracture characteristics.

## Figures and Tables

**Table 1:** The recipe of the PLA-g-GMA graft copolymers.

Samples	PLA (phr)	GMA (phr)	St (phr)	AMS (phr)	EP (phr)	DCP (phr)
A0	100	3	0	0	0	0.15
A1	100	3	6	0	0	0.15
A2	100	3	0	6	0	0.15
A3	100	3	0	0	6	0.15

**Table 2:** The composition of the PLA/PPC/PLA-g-GMA blends.

Samples	PLA (phr)	PPC (phr)	PLA-g-GMA (phr)
B1	70	30	0
B2	65	30	5
B3	60	30	10
B4	55	30	15
B5	50	30	20

**Table 3:** The composition of the PLA/PPC/PLA-g-GMA blends (PLA-g-GMA contained different comonomers).

Samples	PLA (phr)	PPC (phr)	PLA-g-GMA (phr)	PLA-g-GMA/St (phr)	PLA-g-GMA/AMS (phr)	PLA-g-GMA/EP (phr)
G0	100	0	0	0	0	0
G1	0	100	0	0	0	0
G2	70	30	0	0	0	0
G3	60	30	10	0	0	0
G4	60	30	0	10	0	0
G5	60	30	0	0	10	0
G6	60	30	0	0	0	10

**Table 4:** Grafting degree (G_d_) and grafting efficiency (G_e_) of PLA-g-GMA graft copolymers.

Sample	PLA-g-GMA	PLA-g-GMA/St	PLA-g-GMA/AMS	PLA-g-GMA/EP
G_d_ (phr)	0.8	1.6	1.3	0.9
G_e_ (%)	26	53.3	43	30

**Table 5:** Effect of PLA-g-GMA content on mechanical properties of PLA/PPC/PLA-g-GMA blends (G0: PLA; B1: PLA/PPC; B2: PLA/PPC/PLA-g-GMA (5 phr); B3: PLA/PPC/PLA-g-GMA (10 phr); B4: PLA/PPC/PLA-g-GMA (15 phr); B5: PLA/PPC/PLA-g-GMA (20 phr)).

Samples	Elongation at Break (%)	Tensile Strength (MPa)	Notched Impact Strength (J/m^2^)
G0	29.58 ± 4.25	65.19 ± 1.53	3315.4 ± 69.15
B1	108.33 ± 6.14	49.64 ± 1.36	4505.8 ± 80.10
B2	180.00 ± 8.25	48.96 ± 1.44	5050.0 ± 84.14
B3	236.46 ± 10.15	47.38 ± 1.55	5876.9 ± 90.25
B4	197.60 ± 8.34	49.40 ± 1.69	5583.1 ± 85.36
B5	200.31 ± 9.81	48.42 ± 2.88	5623.8 ± 81.91

**Table 6:** Thermal properties of PLA, PPC, PLA/PPC and PLA/PPC/PLA-g-GMA blends.

Samples	T_g_ (PLA)/°C	T_g_ (PPC)/°C	ΔT_g_)/°C	T_m_)/°C	X_c_/%
G0	61.01 ± 0.30	-	-	167.30± 0.20	7.31 ± 0.21
G1	-	30.51± 0.21	-	-	-
G2	59.01 ± 0.25	36.03 ± 0.23	22.98 ± 0.05	167.75 ± 0.11	7.49 ± 0.22
G3	58.80 ± 0.28	36.75 ± 0.27	22.05 ± 0.07	167.59 ± 0.15	9.17 ± 0.37
G4	57.67 ± 0.19	37.25 ± 0.15	20.42 ± 0.05	157.48 ± 0.21	3.94 ± 0.42
G5	57.69 ± 0.31	36.73 ± 0.23	20.96 ± 0.03	162.95 ± 0.20	1.69 ± 0.15
G6	56.51 ± 0.23	35.46 ± 0.42	21.05 ± 0.02	155.19 ± 0.13	0.26 ± 0.21

**Table 7:** Thermogravimetry data of PLA, PPC, PLA/PPC and the PLA/PPC/PLA-g-GMA blends.

Samples	T_95%_ (℃)	T_max_ (℃)	T_5%_ (℃)
G0	303.19 ± 0.12	325.10 ± 0.21	328.08 ± 0.24
G1	273.32 ± 0.23	286.48 ± 0.19	342.94 ± 0.19
G2	277.75 ± 0.14	310.25 ± 0.23	327.60 ± 0.28
G3	269.86 ± 0.21	293.88 ± 0.19	320.85 ± 0.17
G4	276.29 ± 0.20	304.42 ± 0.15	326.53 ± 0.22
G5	276.45 ± 0.17	307.75 ± 0.18	323.02 ± 0.45
G6	270.31 ± 0.09	298.45 ± 0.26	320.38 ± 0.36

Note: T_95%_ is the temperature at 5% loss of specimen mass, T_max_ is the temperature at the maximum rate of specimen weight loss, and T_5%_ is the temperature at 95% loss of specimen mass.

**Table 8:** Effect of grafted monomers on the mechanical properties of PLA/PPC/PLA-g-GMA blends (G0: PLA; G1: PPC; G2: PLA/PPC; G3: PLA/PPC/PLA-g-GMA; G4: PLA/PPC/PLA-g-GMA/St; G5: PLA/PPC/PLA-g-GMA/AMS; G6: PLA/PPC/PLA-g-GMA/EP).

Samples	Elongation at Break (%)	Tensile Strength (MPa)	Notched Impact Strength (J/m^2^)
G0	29.58 ± 4.25	65.19 ± 1.53	3315.4 ± 69.15
G1	761.32 ± 8.69	12.14 ± 1.62	5950.0 ± 85.27
G2	108.33 ± 6.14	49.64 ± 1.36	4505.8 ± 80.10
G3	236.46 ± 10.15	47.38 ± 1.55	5876.9 ± 90.25
G4	286.35 ± 9.25	49.24 ± 1.93	6858.6 ± 98.05
G5	271.67 ± 11.58	47.52 ± 2.84	6686.5 ± 96.47
G6	221.25 ± 8.98	49.96 ± 2.11	5944.3 ± 82.25

## Data Availability

The data presented in this study are available on request from the corresponding author.
